# Strengthening Virology Research in the Association of Southeast Asian Nations: Preparing for Future Pandemics

**DOI:** 10.4269/ajtmh.21-0589

**Published:** 2021-09-10

**Authors:** Adriana Viola Miranda, Lowilius Wiyono, Ian Christopher N. Rocha, Trisha Denise D. Cedeño, Don Eliseo Lucero-Prisno

**Affiliations:** ^1^ Faculty of Medicine, Universitas Indonesia, Jakarta, Indonesia;; ^2^ School of Medicine, Centro Escolar University, Manila, Philippines;; ^3^ Department of Global Health and Development, London School of Hygiene and Tropical Medicine, London, United Kingdom;; ^4^ Faculty of Management and Development Studies, University of the Philippines Open University, Los Baños, Laguna, Philippines

## Abstract

The Association of Southeast Asian Nations (ASEAN) region is known to be a global hotspot to viral outbreaks because of many factors. To limit the impact of future outbreaks, it is crucial for the ASEAN governments to strengthen regional virology research capacity. The ASEAN governments have collaborated in several virology initiatives, with the most recent being the establishment of the ASEAN Regional Center for Public Health Emergencies and Emerging Diseases. However, several challenges, including technology disparities, nationalistic tendencies, and the lack of public acceptance toward virus sharing, need to be addressed to maximize the region’s collaboration potential in virology research. We recommend the governments to 1) prioritize the strengthening of research capacities; 2) develop stronger cooperation and possible centralization of efforts on top of national capacities; 3) develop an equitable and secure research framework; and 4) improve the public awareness regarding the importance of regional public health responses.

## INTRODUCTION

The coronavirus disease 2019 (COVID-19) pandemic has greatly affected all member-countries of the Association of Southeast Asian Nations (ASEAN), with Indonesia and the Philippines being the hardest hit based on the reported number of COVID-19 cases and deaths. The ASEAN countries have been struggling with their national healthcare systems and capacities are unable to keep up with the exponential increase of COVID-19 cases.^1^ As of May 2, 2021, there are 3,352,917 confirmed cases of COVID-19 in the whole region, wherein Indonesia and the Philippines recorded 49.89% and 31.22% of the total cases, respectively.^2^

The COVID-19 pandemic may not come as a surprise as the ASEAN region has been a usual origin of lethal disease outbreaks over the years and, thus, has been recognized as a hotspot for emerging infectious diseases.^3^ This is aggravated by its proximity to southern China which is part of the hotspot corridor. In 1998, Malaysia had an outbreak of Nipah virus, which resulted in more than 250 human cases and more than 100 deaths. In 2003, an outbreak of severe acute respiratory syndrome (SARS) occurred in Singapore and Vietnam, resulting in 63 cases with five deaths and 238 cases with 33 deaths, respectively. In 2004, the H5N1 avian influenza reached Cambodia, Indonesia, Laos, Thailand, and Vietnam with over 350 human cases and 250 deaths in the region despite attempts to contain the virus by culling infected chickens and ducks. In 2009, the influenza A(H1N1) pandemic also reached the region, infecting more than 55,000 people with more than 300 deaths.^4^ And recently, in 2020, COVID-19 entered the ASEAN region. In fact, the first reported case of COVID-19 outside China was identified in Thailand and the first COVID-19-related death outside China was reported in the Philippines.^2^ Given this risk profile, it is surmised that many viruses originate from the ASEAN region, which is attributed to a number of factors.

The ASEAN region provides a profile where dynamic structures coexist and interact. These include biological, social, and environmental systems, which interact in ways that support microbes to develop and grow in new habitats. The growing population and migration within the region, deforestation and industrialization, developments in food production, cultivation and land utilization, water and sanitation, and the impact of healthcare systems through drug resistance are examples of these processes. Deforestation in Malaysia, for example, has resulted in the destruction of the natural habitat of fruit bats, which is linked to the emergence of the Nipah virus.^4^ As humans cause deforestation, it affects the environment of animals that are usually hosts of viruses, resulting in an increase in the number of viruses that can cause outbreaks within the region. The exposure of wild animals to human populations increases the movement and jumping of viruses.

With this profile as a global hotspot of outbreaks, the COVID-19 pandemic is a reminder that there would always be room for improvement when it comes to disaster risk reduction and preparedness—and it stems from having a strategic and organized plan on public health responses. Seeing a strong linkage between the occurrence, reemergence, and/or spread of several viral infections among ASEAN countries to their sociocultural upbringing, it is therefore vital to pay more attention to strengthen virology research in the ASEAN region.

## CURRENT EFFORTS

Recognizing the importance of regional public health cooperation in the context of shared public health threats, ASEAN governments have launched several ASEAN-wide initiatives. In response to the COVID-19 pandemic and previous outbreaks, the ASEAN exchanged laboratory readiness and response through training conducted by the Regional Public Health Laboratory (RPHL) led by Thailand.^5^ The ASEAN Secretariat also announced plans to establish the ASEAN Regional Center for Public Health Emergencies and Emerging Diseases (ACPHEED), which has the following roles:

1. Facilitate the development of joint regional surveillance and laboratory responses,
2. Receive initial public health emergency report throughout the ASEAN region,
3. Coordinate with the ASEAN Secretariat and other relevant bodies to facilitate intersectoral coordination,
4. Serve as the repository of information regarding public health emergencies of international concern,
5. Prepare periodic reports, and
6. Research and develop prevention, surveillance, and response initiatives.^6^

The ASEAN member countries have pledged funding to the center. Japan has also given funding support. Currently, Indonesia, Thailand, and Vietnam are bidding to host the center. Upon establishment, ACPHEED will primarily serve as a facilitator of joint surveillance and research responses upon requests from ASEAN member countries. The member countries are expected to provide local logistical arrangements in accordance with a predetermined requirement, with funding assistance from the ASEAN Secretariat. The ACPHEED will take a more active role if the WHO announces a Public Health Emergency of International Concern (PHEIC), during which the ACPHEED will collaborate with WHO and coordinate responses through the ASEAN Secretariat.^6^

Considering the previous success of the Southeast Asia Infectious Disease Clinical Research Network (SEAICRN), a collaborative partnership between Thailand, Vietnam, and Indonesia, the ACPHEED establishment is expected to accelerate the advancement of virology research in the region. The SEAICRN, established in 2005, has supported about 100 infectious diseases research projects throughout the region, many of which are multicenter studies.^7^ The establishment of ACPHEED will not only extend the benefit to other ASEAN countries, but also enable the translation into public policy findings of multicenter studies. It is currently unknown whether SEAICRN and ACPHEED will collaborate in the future, however, both centers are expected to be exclusive of each other with their respective focus of program in achieving strengthened virology research and mitigation plan.

## CHALLENGES AND RECOMMENDATIONS

Considering the high mobility, as an offshoot of the ASEAN free mobility framework,^8^ and potential transmission of infectious diseases across the region, there is a need to develop stronger cooperation between ASEAN countries. Centralization of regional virology research efforts will be of high benefit to all as it will be an immediate response at any geographical location.^9^ The centralized and regional approach has been effective in other regions like the African Union (AU). The Africa Centers for Disease Control and Prevention was formalized and established by the AU in 2017 after the 2014 Ebola outbreak. Currently, the organization’s Institute of Pathology Genomics leads the COVID-19 variants sequencing in the region and facilitates joint surveillance.^10^

Based on ASEAN geographic position and key industrial sector, the virology research focuses in the ASEAN should include 1) the detection and diagnostic technologies for virus infections in agricultural and horticultural products (zoonoses); 2) the discovery of biomarkers, target molecules for the diagnosis and treatment of viruses endemic in the ASEAN; and 3) surveillance and preventive measures to control outbreaks. Early outbreak identification will help reduce the number of regional epidemics that can lead to pandemics. It is recommended that ACPHEED and other joint response programs serve not only to facilitate such responses, but also as the central command of these operations.

The main challenge of this centralized research collaboration in ASEAN is the huge disparity of laboratory capacities among its member states. While Singapore has the most advanced research capacity, Myanmar, Laos, and Cambodia are still struggling to provide basic laboratory services like HIV/AIDS and hepatitis B. Several large institutions in Malaysia, Thailand, and Indonesia are equipped with sophisticated molecular biology tests, with lesser availability in Indonesia.^11^ Even with the RPHL and ACPHEED facilitating joint laboratory responses, this disparity limits the types of virology research that can be pursued.

These differences in available technology, resources, and capacities have also been proven to impact the preparedness to conduct virology research in these countries. Although most have learned and fought the influenza in the last few decades, only some have truly taken the advantage of their experience as exemplified by Singapore through its National Center for Infectious Disease and by the Philippines with its Research Institute for Tropical Medicine.^12^^,^^13^ Other ASEAN countries, in particular, Vietnam and Thailand, have applied their capabilities to conduct virology research on the race for COVID-19 management, with their own vaccine production, such as Nanocovax from Vietnam and AstraZeneca production plan in Thailand.^14^ To ensure stronger resilience toward future pandemics, the ASEAN governments need to invest more in research capacity, including equipment, technology, and personnel training. It is also recommended that the higher-income countries in the region provide funding support to their lower-income neighbors.

The potential nationalistic tendencies during public health responses may also threaten the effectiveness of the ASEAN-based response. The first major incident in the ASEAN happened back in 2005, when Indonesia stopped sharing influenza virus samples to the WHO, as required by the International Health Regulations for global surveillance and responses. The country, with support from other countries, protested the unfair vaccine allocation experienced by the poorer countries, which comprises many of the virus donors.^15^ Although this concern is not unfounded and resulted in more equitable vaccine distribution, this incident indicates that such disruption may occur again in the future. There is also a growing concern that virus sharing can lead to the development of biological warfare by countries with more advanced research capacity.^16^ This may reduce the public support toward the regional virology research cooperation, thus lessening the participation of countries that are crucial for the success of the program. Therefore, it is argued that ASEAN stakeholders develop an equitable and more secure research framework.

Improving public awareness on the importance of regional public health responses is also crucial for the success of ASEAN virology research strengthening. With proper planning and adequate funding, collaboration with civil society organizations (CSOs) can ensure public acceptance and involvement in the centralized virology research scheme because CSOs are experienced in engaging their respective communities.^17^

## CONCLUSION

Strengthening ASEAN virology research capacity is crucial to support the region’s public health preparedness. Recognizing this importance, the ASEAN governments have established several virology research initiatives. However, more efforts are needed to ensure their success: the governments need to develop a stronger regional collaboration and centralization of research efforts, invest more in research advancement, develop an equitable and secure research framework, and improve the public awareness regarding the importance of such initiatives.
